# Construction of Cognitive Maps to Improve Reading Performance by Text Signaling: Reading Text on Paper Compared to on Screen

**DOI:** 10.3389/fpsyg.2020.571957

**Published:** 2020-09-30

**Authors:** Zifu Shi, Ting Tang, Lin Yin

**Affiliations:** ^1^Department of Psychology, School of Educational Science, Hunan Normal University, Changsha, China; ^2^Cognition and Human Behavior Key Laboratory of Hunan Province, Hunan Normal University, Changsha, China; ^3^Hunan Normal University Library, Hunan Normal University, Changsha, China

**Keywords:** physical signaling, verbal signaling, cognitive map, on-screen reading, paper reading, navigation

## Abstract

Reading text from a screen has been shown to be less effective compared with reading text from paper. Various signals may provide both background information and navigational cues, and may promote the construction of cognitive maps during on-screen reading, thus improving reading performance. This study randomly divided 75 college students into a paper reading group and an on-screen reading group. Both groups were tested for navigation and reading comprehension in response to three different forms of signaling (plain text, physical signaling, and verbal signaling). The results showed that when plain text was presented, the navigation and comprehension scores of the paper reading group were significantly higher than those of the on-screen reading group. However, no significant difference was found between both groups under signaling conditions. The navigation and comprehension scores of both groups were significantly higher under signaling conditions than under plain text. Moreover, the comprehension score of the on-screen reading group under physical signaling was significantly higher than that under verbal signaling. This research suggested that signals help to construct cognitive maps and effectively improve reading performance. Besides, physical signaling, such as underlining and bold formatting, is more effective for on-screen reading. The present study provides a practical and effective approach for improving on-screen reading based on cognitive map theory.

## Introduction

As a result of the development of electronic technologies, reading has become increasingly digitized. The popularity of reading on digital reading devices, such as iPad and Kindle, has significantly decreased visual fatigue and operational discomfort during on-screen reading (Lin et al., 2008). Therefore, readers are satisfied with such on-screen reading devices for reading prose, which does not require the application of active reading strategies (Thayer et al., 2011). However, on-screen reading seems to be only appropriate for reading prose, such as novels. When reading more complicated and challenging texts such as expository text or technical content, on-screen reading remains insufficient and has been shown to be unsuitable (Liu, 2005; Clinton, 2019). Therefore, this study used expository text as research material to explore how the on-screen reading performance of such texts can be more effectively improved.

### Cognitive Map

The idea of a cognitive map originates from the theoretical research of psychology on spatial cognition. It is a cognition form that represents environmental information, and it is a similar model to the field map formed in the brain based on past experience (Yang and Bi, 2005). When the human brain collects visual information about an object, it also collects information about its surroundings and connects them together (Jabr, 2013; Li et al., 2013). As a result, when people read a text, not only the words and semantics of the text but also the physical location and background information of the text enter the brain for processing as a whole, forming a cognitive map of the text (Payne and Reader, 2006; Hou et al., 2017a). Similar to how a physical landscape is remembered, readers form a cognitive map of the physical location of text segments on a page (Hou et al., 2017b). During the reading process, readers first identify “landmarks,” namely, important concepts, knowledge, or information. Then, they construct routes between the landmarks, i.e., front and back, far and near, as well as hierarchical relationships between concepts, knowledge, or information in logical and spatial positions. Finally, they integrate these landmarks and relationships into survey knowledge, i.e., build textual cognitive maps (Foo et al., 2005; Voeroes et al., 2011). Based on this, cognitive maps in the reading area can be identified as the mental representation of the structure of a text and its background context that are constructed by readers during reading (Thayer et al., 2011; Li et al., 2013; Hou et al., 2017a,b). The construction of such cognitive maps not only helps to locate the content that has been read, but also leads to more effective retention and recall of text information (Rothkopf, 1971; Lovelace and Southall, 1983; O’Hara et al., 1999; Morineau et al., 2005).

According to cognitive map theory, whether a text presentation can promote the formation of a cognitive map of the text structure is the key factor that influences reading outcomes (Hou et al., 2017a,b). During paper reading, the provision of rich background information helps the formation of knowledge landmarks (Li et al., 2016), which readers can use to locate information and associate its physical position in the text with the logical order of its contents (Li et al., 2013; Mangen et al., 2019), thus forming survey knowledge. However, because of the lack of background information and navigational cues during on-screen reading (Jabr, 2013; Li et al., 2013) as well as the loss of spatial knowledge about the location of specific content, readers are unable to attain an overall grasp of the text structure, which thus obstructs their construction of an effective mental map (Morineau et al., 2005; Payne and Reader, 2006; Rose, 2011; Thayer et al., 2011).

Consequently, an important question is how on-screen text can be better displayed to help readers construct cognitive maps and thus improve their on-screen reading performance. Li et al. (2013) developed an e-reader that combines maps of visual cues and two reading strategies and found that maps of visual cues can help readers to construct cognitive maps during on-screen reading, which promotes navigation and reading comprehension. Hou et al. (2017a) suggested that as long as the text presentation for on-screen reading completely imitates that for paper reading, it is conducive to the construction of cognitive maps, and readers’ performance during on-screen reading tasks can be improved. Tang et al. (2020) designed an iReader digital reading program that is equivalent to paper text for cognitive map construction conditions. They found that under these specific conditions, no difference was found between on-screen and paper reading performances. These studies indicated that as long as the conditions for the construction of cognitive maps are similar to those for paper texts, the on-screen reading performance can be improved. However, these studies mainly help readers to construct cognitive maps and improve their on-screen reading performance by utilizing reading software and technology. Since cognitive maps are formed during the processing of textual information by a reader, it is possible to identify effective ways to construct cognitive maps based on reading behaviors and habits. The research on signals inspired our discussion of this issue.

### Signals

In the process of reading, to effectively complete the reading task, learners usually adopt certain reading strategies to master the content of reading materials and solve problems in reading. Text signaling is one of the most used reading strategies (Li et al., 2016). Text signals include words, phrases, sentences, or special symbols that can appear in different places within a text, but rather than adding any new content, they emphasize the structure or specific content of the text (Britton et al., 1982; Lorch, 1989; Van Gog, 2014). The signaling promotion effect is defined as the promotion effect of text signals on comprehension processes and information retention of a text (Lorch et al., 1993; Lorch and Lorch, 1996; He and Mo, 2000). In multimedia learning, it is also known as the signaling principle or cueing principle, and it refers to the finding that people learn better when signals are added that guide attention to certain elements of the material or highlight the structure (Mayer, 2005; Van Gog, 2014). Signaling forms mainly consist of physical signaling and verbal signaling. Physical signaling is defined as emphasizing important information and words mainly by highlighting, underlining, and bold formatting. Verbal signaling includes headings, summaries, and organizing charts (He and Mo, 2000; Mayer, 2005). Organizing charts for verbal signaling utilize a visual method to analyze and compare keywords, concepts, or central sentences within a text, determine their hierarchical relationship, and present the main structural framework of the text in the form of a network (Du et al., 2006). It has been reported that organizing charts help readers to organize and represent knowledge (Hagemans et al., 2013), which improves their level of recall and comprehension of the reading material (DeLauder and Muilenburg, 2012).

During the early stage, research on the promotion effect of signals was mainly conducted in the form of experiments to test the impact of specific signals on paper reading. Most relevant studies suggested that readers could achieve better recall performance and reading comprehension for texts that include signals compared with texts that are devoid of signals (Johnson, 1988; Lorch et al., 1993; Amer, 1994; Lorch and Lorch, 1996). With the advent and increasing popularity of the Internet after the year 2000, an increasing number of scholars have voiced concern about the role of signals in other reading media besides paper, focusing on hypertext and hypermedia learning. Many studies found that proper inclusion of cues in multimedia learning materials can help to improve the academic performance of readers (de Koning et al., 2007, 2010; Mautone and Mayer, 2007). For example, Jamet (2014) confirmed that the integration of signals is conducive to promoting the integrated processing of graphics and text, and significantly improves the test scores of learners. Colliot and Jamet (2018) also found that the use of outlines as signals promotes the memorization and comprehension of learning materials by college students and enables them to achieve higher scores in both retention and transfer tests. Although many studies have shown that signals cannot improve learning performance (Lowe and Boucheix, 2011; Li et al., 2016), eye movement experiments by Kriz and Hegarty (2007) showed that cues can effectively guide learners to notice task-related information, while not enabling them to achieve higher scores in retention and transfer tests. However, most studies suggested that signals can guide the attention distribution of readers, provide cues that are important for the reading processing, help readers to form a representation of the organizing chart of the article, and ultimately promote both reading comprehension and retention (Lorch et al., 1993; Lorch and Lorch, 1996; Mautone and Mayer, 2001; Ponce and Mayer, 2014).

In summary, existing research has shown that on-screen reading is not as effective as paper, especially for expository texts where the purpose is to give information and there is a need for a deeper and more detailed level of processing (Margolin et al., 2018). Researchers suggested that this is because it is difficult to construct effective cognitive maps with on-screen readers, but cognitive map theory is rarely studied empirically. Meanwhile, it was found that the use of signals can promote the efficiency of both paper reading and multimedia learning, and can provide cues that help readers to construct structural representations of the subject of the text. However, in on-screen reading, whether the presented signals have the same effect they have in paper reading still requires further investigation. Few studies have explained how different signals affect the on-screen reading. Therefore, the present study compared reading and navigation performance with on-screen and paper reading to examine the cognitive map theory of on-screen reading. Besides, we assumed that on-screen signals can provide readers with background information and navigation cues, and can thus help readers to construct cognitive maps when reading on-screen text, thus effectively improving navigation and reading comprehension. At the same time, because of the structural integrity and visual intuition of the utilized organizing chart, an organizing chart may be more conducive to the formation of cognitive maps and thus, the improvement of reading performance than the use of bold formatting and underling. To this end, this study designed experiments to specifically investigate the impact of different forms of signaling on navigation and reading comprehension on different media. Furthermore, their internal mechanisms were investigated by the combination of signal and cognitive map theory. This study attempted to answer three research questions:

1. Is on-screen reading not as effective as paper reading for expository text?
2. Does text signaling helps to construct cognitive maps during on-screen reading, thus improving both navigation and reading comprehension?
3. Is verbal signaling (organizing charts) more helpful for the construction of cognitive maps and does it improve reading performance more than physical signaling (bold formatting and underling) for on-screen reading?

## Materials and Methods

### Participants

Seventy-five freshmen students (mean age 19.53 ± 1.39, 52 male, and 23 female) were recruited from two classes majoring in electronic science and technology at Central South University of China. Their majors were consistent, thus avoiding the impact of their professional background on reading comprehension and navigation. All the participants had normal or corrected eyesight and no dyslexia. Before the reading session, participants completed a pre-test questionnaire asking about their demographic information, such as sex, age, Chinese language scores, and their on-screen reading experience and screen using habits. It was found that all the participants had the necessary language reading ability to participate in the experiment (their average score of the Chinese language for the college entrance examination was 112.56, *SD* = 7.41 and scores ranged from 97 to 130, with a total score of 150 and the passing score is 90). These participants all used or had exposure to electronic screens very often in their daily life and they averaged 2.74 h of text reading on electronic devices per day (*SD* = 1.50), and, consequently, they were considered to be familiar with on-screen reading.

Half of the students were assigned to the paper reading group, and half were assigned to the on-screen reading group with the gender approximately balanced across the groups. See Table 1 for a summary of the participants and pre-testing details. The equivalence of the demographic variables, screen using habits, and on-screen reading experience between groups were tested using a series of one-way ANOVA. No significant difference was found on these pre-test scores of the two groups (*p* < 0.05). Thus, the groups were equivalent in terms of these variables (e.g., age, Chinese language scores, on-screen reading experience, and screen using habits). After reading, the experimenter checked with the participants if they had read the expository texts or acquired relevant knowledge before. This was not the case for any of them. The study had prior approval by the Ethics Committee of Hunan Normal University in China. We obtained written informed consent from all the participants, and each of them was paid 20 RMB for participating.

**Table 1:** Group characteristics.

Group	Sample	Age (years) *M (SD)*	Chinese language scores *M (SD)*	On-screen reading experience^a^ (h) *M (SD)*	Screen using habit^b^ *M (SD)*
Paper	*n* = 38 (12 females)	19.53 (0.83)	113.47 (7.44)	3.08 (1.55)	3.11 (0.56)
On-screen	*n* = 37 (11 females)	19.54 (1.80)	111.62 (7.37)	2.39 (1.37)	3.22 (0.58)

a On-screen reading experience: hours of reading text on screen per day.

b Screen using habits (how often do you use or exposure to electronic screens in daily life): rarely or never use = 1, occasionally use = 2, often use = 3, and always use = 4.

### Materials

Before the experiment started, pre-tests were conducted, and three expository texts were selected as reading materials. Ten Chinese technical expository texts were selected from the “Civil Servants Exam 2018: 200 Articles.” The original text and corresponding test questions were partially modified to be more in line with the experimental requirements regarding length, language expression, and question types. Each text contained 1,200–1,300 words and was displayed on two pages. Twenty college students were selected to take pre-tests on these 10 expository texts, each of which was asked to read the 10 texts, complete the corresponding test questions, and select the final three articles with similar test scores and medium difficulty as reading materials. The contents of these three texts involved the “origin of civilization,” “energy and economy,” and “food additives,” respectively.

**Figure 1:**
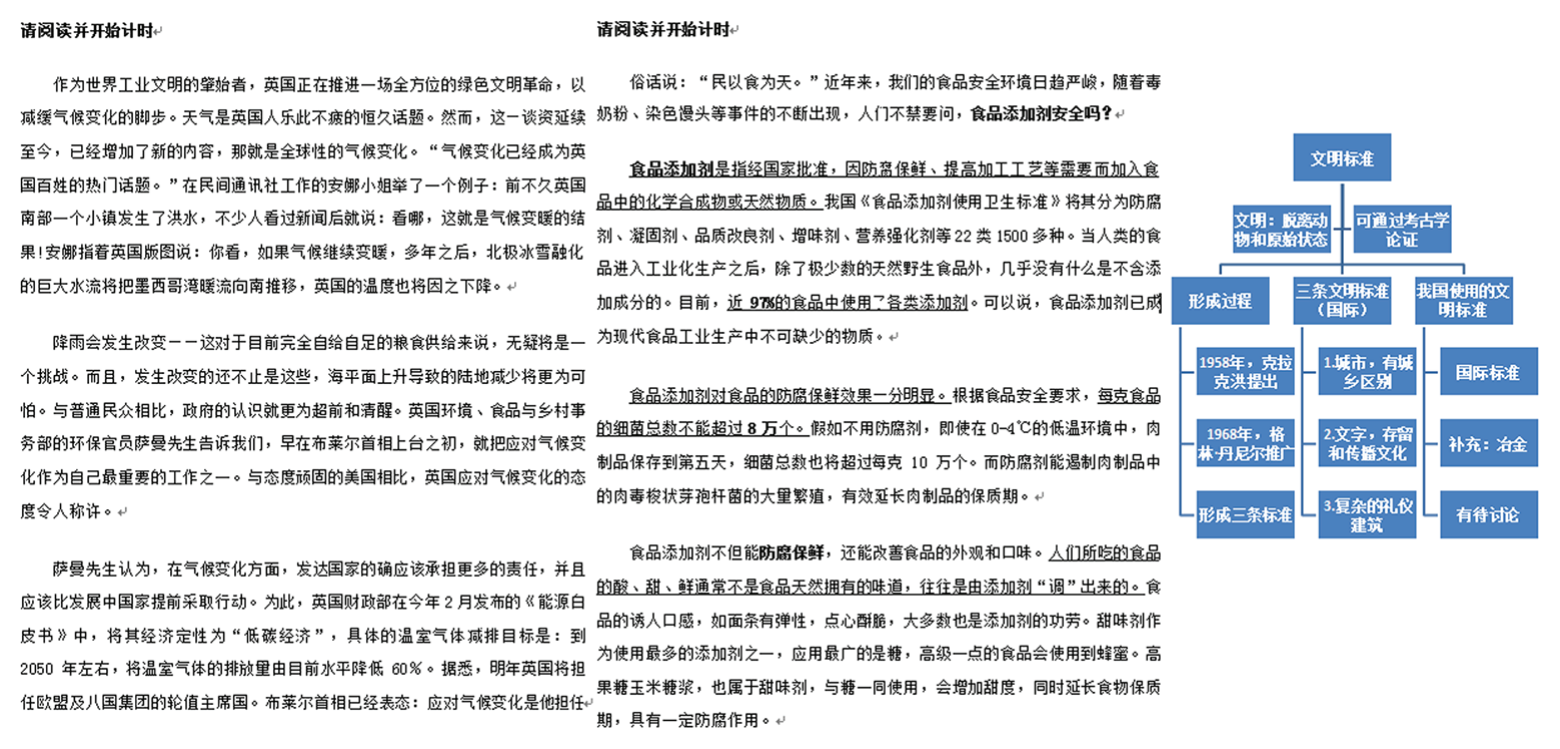
Three forms of signaling. The left-hand page shows plain text (text without any signals). The middle page shows physical signaling (text with underlining and bold formatting). The right-hand page shows verbal signaling (an organizing chart of the text which was presented at the end of the text).

Two experienced professional tutors processed the plain text and added physical signaling and verbal signaling to the three scientific expository texts (see Figure 1). Plain text (also named non-signaling text) refers to text with neither physical signaling nor verbal signaling information. Physical signaling indicates that key concepts and sentences in the text are underlined or formatted in bold. Specifically, we bolded the core concepts and key points of the expository text and underlined the topic sentences and summary sentences (e.g., in the text about “food additives,” the first sentence of the second paragraph “Food additives refer to chemical compounds or natural substances that have been approved by the state to be added to food for anti-corrosion and freshness preservation, improvement of processing technology, etc.” was underlined, and “food additives” was formatted in bold). Verbal signaling indicates that the text was presented together with an organizing chart, which shows key items and their relationships in the text (e.g., in the organizing chart based on the text about “origin of civilization,” the key items include: “Civilization,” “Three International Civilization Standards,” and “Civilization Standards Used in our country,” etc.; Hagemans et al., 2013).

### Reading Comprehension Test

There were eight reading comprehension questions after each text, four judgment questions, and four single choice questions to assess two specific aspects of detailed recall and comprehension inference. Among these questions, the first and second judgment questions and the seventh and eighth single choice question were inference questions, which were used to examine the readers’ comprehension and inference ability to understand the overall meaning of the text. A sample item of a judgment question is: “Based on the meaning of the text, nations without cities have not entered the stage of civilization.” The third and fourth judgment questions and the fifth and sixth single choice questions were recall questions, which tested the readers’ ability to recall text details. A sample item of a single choice question is: “Why does the UK Treasury provide interest-free loans to some enterprises?” For each question, one correct answer scored one point, one incorrect answer scored zero points, and each text totaled eight points.

### Navigation Test

According to the measurement methods used by Mangen et al. (2019), participants were asked to locate four related contents or concepts in the text, which were placed either in the first half of the first page, the second half of the first page, the first half of the second page, and the second half of the second page (sample item: “Please locate the following contents to their correct place in the text: In which year was the Initial Civilization published?”). For each question, one correct answer scored one point, one incorrect answer scored zero points, and each text totaled four points.

### Experiment Design and Apparatus

This study used a two-way mixed experimental design of 2 (media: paper vs. on-screen) × 3 (signaling: plain text vs. physical signaling vs. verbal signaling) with media as the between-participants variable and signaling as the within-participants variable. The paper group read the texts on printed A4 paper, and the on-screen group read texts on a 19-in DELL screen (using Microsoft Word 2010 software). The text content, layout, format, color, and display form both media used for the presentation were identical. The reading and navigation performance of the participants was the dependent variable. The reading performance was measured *via* reading comprehension scores (the sum of the scores of recall and inference questions), while the navigation performance was measured *via* navigation test scores.

### Procedure

The experiment was conducted in a usability laboratory. First, participants were introduced to the experiment and finished the consent procedure. Subsequently, they completed a pre-test, that is, a paper-and-pen questionnaire asking about their demographic information and on-screen reading habits and experiences. Then, participants were assigned to the paper reading group and the on-screen reading group and were instructed to read the texts at their normal pace. They were not informed of the exact purpose of the experiment but only that they were going to read three texts in different signaling forms on a computer or on paper and that they would answer some questions afterwards. The experimenter recorded the reading time.

After reading, participants first completed the corresponding comprehension tests and navigation tests for each text before continuing to the next text. All participants conducted paper-and-pen tests without a time limit, and they were not allowed to look back at the materials when answering the questions. To control for the order error, the order of the three texts in different signaling forms was randomized. The three texts were arranged into six sequences (e.g., abc, acb, bac, bca, cab, and cba). According to the number of participants in the two groups (paper reading group: 38 and on-screen reading group: 37), every six participants were assigned to one of the six sequences, and the remaining one or two participants were randomly assigned to any one of the six sequences. The experiment lasted approximately 10–20 min.

### Data Analysis

Data were analyzed using SPSS software. The data analysis of this study mainly included four parts: (1) descriptive statistics and a one-way ANOVA of reading time; (2) descriptive statistics on the results of total comprehension and navigational performances; (3) two-way repeated measures ANOVA of 2 (media: paper and on-screen) × 3 (signaling form: non-signaling, physical signaling, and verbal signaling) performed *via* comprehension scores and navigation scores, respectively; and (4) the same repeated measures ANOVA performed on the scores of inference and recall questions, respectively. When the interaction between both independent variables was significant, the simple effect was further analyzed.

## Results

### Reading Time

Participants completed the reading within 185–735 s (i.e., 3.10–12.25 min), *M* = 450.11 (i.e., 7.50 min), and *SD* = 99.75. There was no difference between reading media in terms of reading time [*M*_paper_ = 443.13, *SD* = 82.24; *M*_on-screen_ = 457.27, *SD* = 115.75; *F*(1,73) = 0.373, *p* = 0.543, and partial *η*^2^ = 0.005]. The two groups had the same reading speed in the present experiment according to the result.

### Reading Performance

Descriptive statistics of comprehension scores and navigation scores of paper and on-screen groups under different signaling forms are listed in Table 2.

**Table 2:** Descriptive statistics of dependent variables under different reading conditions *M (SD)*.

	Non-signaling		Physical signaling		Verbal signaling	
	Comprehension scores	Navigation scores	Comprehension scores	Navigation scores	Comprehension scores	Navigation scores
Paper	5.18 (1.45)	2.37 (0.97)	6.61 (0.92)	3.29 (0.77)	6.37 (1.10)	3.03 (0.91)
On-screen	4.41 (1.04)	1.86 (0.95)	6.65 (0.86)	3.22 (0.79)	6.11 (1.02)	2.81 (0.78)

### Comprehension Score

**Figure 2:**
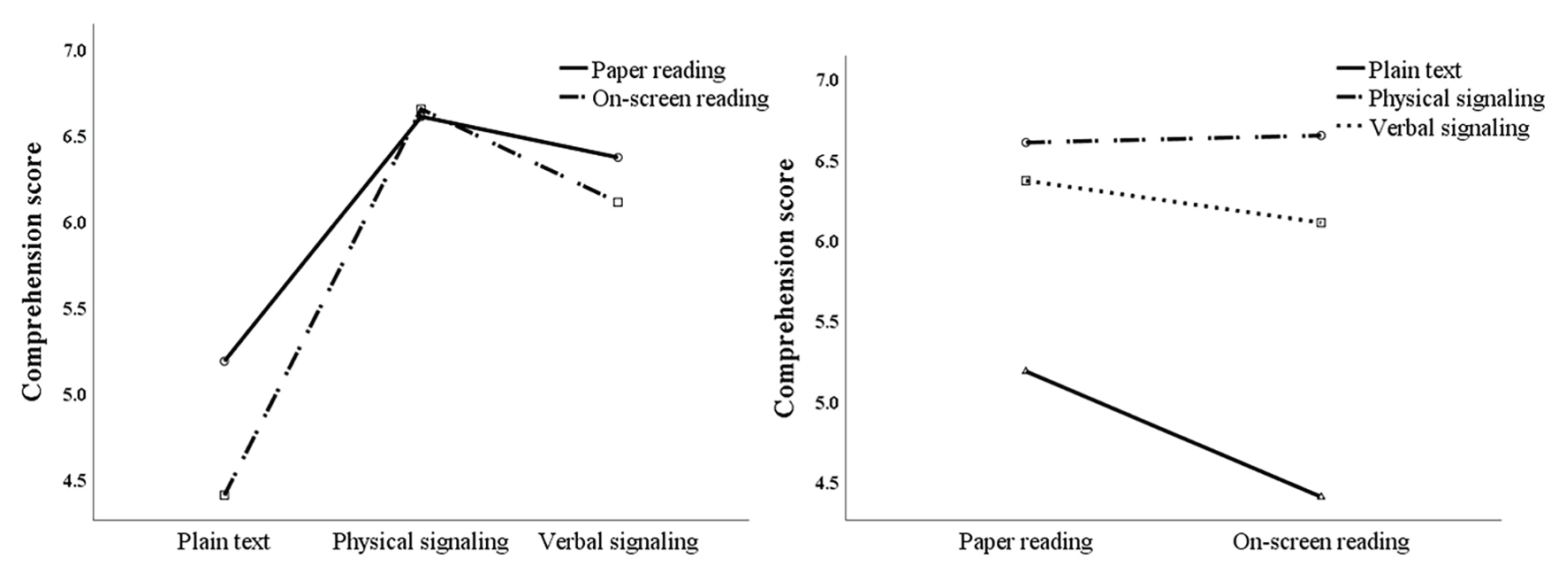
Interaction analysis between reading media and signaling forms for comprehension scores.

Repeated measures ANOVA of comprehension scores showed that the main effect of reading media was significant, *F*(1,73) = 4.462, *p* < 0.05, and partial *η*^2^ = 0.058, the main effect of signaling form was significant, *F*(2,72) = 65.742, *p* < 0.01, and partial *η*^2^ = 0.474, and the interaction between signaling form and media was marginally significant, *F*(2,72) = 3.048, *p* = 0.050, and partial *η*^2^ = 0.040. Further simple effect analysis (see Figure 2) showed that, in a comparison of different reading media, under the non-signaling condition, the comprehension score of the paper reading group was significantly higher than that of the on-screen reading group, *F*(1,73) = 7.117, *p* < 0.01, and partial *η*^2^ = 0.089, while under the physical signaling [*F*(1,73) = 0.045, *p* > 0.05, and partial *η*^2^ = 0.001] and verbal signaling [*F*(1,73) = 1.125, *p* > 0.05, and partial *η*^2^ = 0.015], no significant difference was found between comprehension scores of the paper reading group and the on-screen reading group. Simple effect comparison of different signaling forms showed that under the condition of paper reading, participants’ comprehension scores differed significantly under different signaling forms [*F*(2,72) = 18.553, *p* < 0.01, and partial *η*^2^ = 0.340]. Specifically, no significant difference was found between comprehension scores of physical and verbal signals, but both scores were significantly higher than that of non-signaling. Under the condition of on-screen reading, participants’ comprehension scores were also significantly different under different signaling forms [*F*(2,72) = 43.487, *p* < 0.01, and partial *η*^2^ = 0.547]. Specifically, the comprehension score of physical signaling was significantly higher than that of verbal signaling and non-signaling, and the score of verbal signaling was significantly higher than that of non-signaling.

### Navigation Score

**Figure 3:**
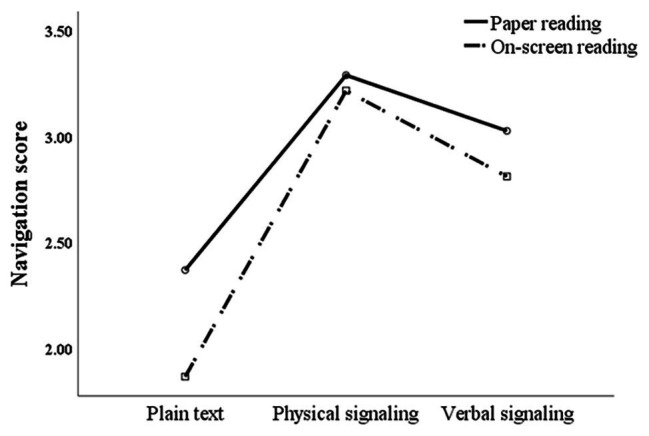
Interaction analysis between reading media and signaling forms for navigation scores.

Repeated measures ANOVA of navigation scores showed that the main effect of the signaling form was significant [*F*(2,72) = 29.238, *p* < 0.01, and partial *η*^2^ = 0.448]. Further pairwise comparison showed that participants’ navigation scores under physical signaling were significantly higher than under the other two signaling forms, and the navigation score under verbal signaling was significantly higher than that under non-signaling. The main effect of reading media was significant [*F*(1,73) = 4.388, *p* < 0.05, and partial *η*^2^ = 0.057]. Specifically, under the non-signaling condition, the navigation score of the paper reading group was significantly higher than that of the on-screen reading group [*F*(1,73) = 5.166, *p* < 0.05, and partial *η*^2^ = 0.066], while in physical signaling [*F*(1,73) = 0.167, *p* > 0.05, and partial *η*^2^ = 0.002] and verbal signaling [*F*(1,73) = 1.207, *p* > 0.05, and partial *η*^2^ = 0.016], no significant difference was found between both groups. There was no significant interaction effect [*F*(2,72) = 1.051, *p* > 0.05, and partial *η*^2^ = 0.028; see Figure 3].

### Inference Score

**Figure 4:**
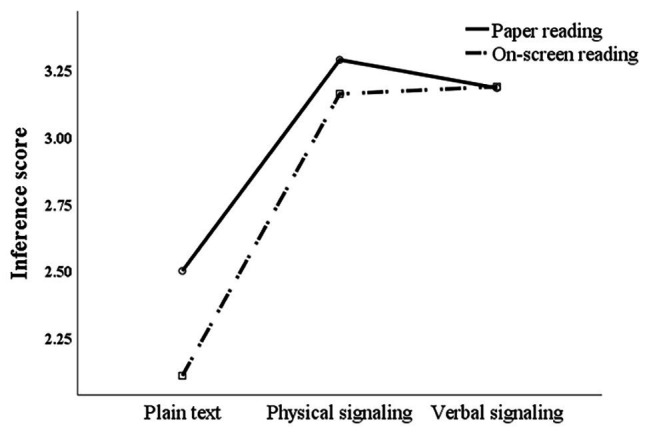
Interaction analysis between reading media and signaling forms for inference scores.

To further clarify the underlying reasons for the effects of physical and verbal signals on comprehension scores in different media, this study analyzed the differences in the comprehension scores of inference and recall questions. Two-way repeated measures ANOVA of the inference score showed that the main effect of the signaling form was significant [*F*(2,72) = 36.918, *p* < 0.01, and partial *η*^2^ = 0.336]. Further pairwise comparison showed no significant difference in comprehension scores between physical and verbal signals, but both scores were significantly higher than under non-signaling. Concerning the reading media, although the inference score of the paper reading group was slightly higher than that of the on-screen reading group, there was no significant difference between the two groups [*F*(1,73) = 2.476, *p* > 0.05, and partial *η*^2^ = 0.033]. There was no significant interaction effect [*F*(2,72) = 1.387, *p* > 0.05, and partial *η*^2^ = 0.019; see Figure 4].

### Recall Score

**Figure 5:**
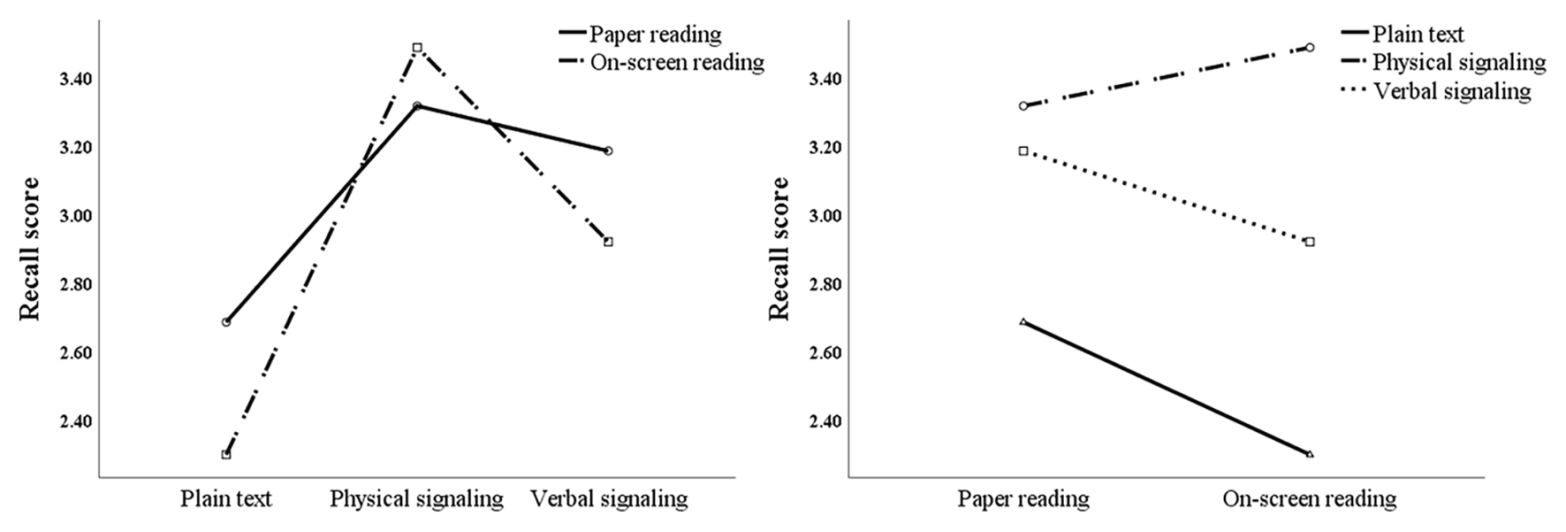
Interaction analysis between reading media and signaling forms for recall scores.

Two-way repeated measures ANOVA of the recall score showed that the main effect of the signaling form was significant [*F*(2,72) = 27.474, *p* < 0.01, and partial *η*^2^ = 0.273], while the main effect of the reading media was not significant [*F*(1,73) = 2.529, *p* > 0.05, and partial *η*^2^ = 0.033]. The interaction between the signaling form and the media was marginally significant [*F*(2,72) = 2.800, *p* = 0.064, and partial *η*^2^ = 0.037; see Figure 5].

A further simple effect analysis showed that in the comparison of different reading media, under the non-signaling condition, the recall score of the paper reading group was significantly higher than that of the on-screen reading group, [*F*(1,73) = 4.459, *p* < 0.05, and partial *η*^2^ = 0.058], while both under the physical signaling [*F*(1,73) = 0.045, *p* > 0.05, and partial *η*^2^ = 0.001] and verbal signaling [*F*(1,73) = 1.125, *p* > 0.05, and partial *η*^2^ = 0.015], no significant differences were found between recall scores of the paper reading group and the on-screen reading group.

Simple effect analysis for the comparison of different signaling forms showed that under the condition of paper reading, participants’ recall scores were significantly different under different signaling forms [*F*(2,72) = 7.129, *p* < 0.01, and partial *η*^2^ = 0.165]. Specifically, there was no significant difference between recall scores of physical and verbal signals, but both scores were significantly higher than that of non-signaling. Under the condition of on-screen reading, participants’ recall scores were also significantly different under different signaling forms [*F*(2,72) = 24.421, *p* < 0.01, and partial *η*^2^ = 0.404]. Specifically, the recall score of physical signaling was significantly higher than that of verbal signaling and non-signaling, and the recall score of verbal signaling was significantly higher than that of non-signaling.

The results of the inference scores were different from the total comprehension scores, while the results of recall scores were similar to the total comprehension scores. This implies that the different reading performances for different media affected by physical signaling and verbal signaling were mainly a result of recall questions.

## Discussion

This study compared the effects of physical signaling and verbal signaling on reading comprehension and navigation when reading expository texts either on-screen or as printed text. An experiment was conducted to answer three research questions. The results showed that the reading comprehension and navigation scores in the case of signaling were significantly higher than those of non-signaling, indicating that signals help to construct cognitive maps during reading, which showed a signaling promotion effect. Moreover, comparing the promotion effect for reading performance between different media of physical and verbal signals showed that for reading on paper, no significant difference was found in the comprehension scores under both forms of signaling; for reading on screens, the comprehension score of physical signaling was significantly higher than that of verbal signaling. This shows that physical signaling can promote on-screen reading more effectively than verbal signaling. The following presents further analysis and discussion.

Regarding question 1, this study showed that during reading non-signaling texts, the comprehension and navigation scores of the paper reading group were significantly higher than those of the on-screen reading group. Therefore, question 1 could be answered, that is, on-screen reading is not as effective as paper reading when reading technical expository text, which is consistent with existing research results (Singer and Alexander, 2017; Clinton, 2019). According to the cognitive maps theory (e.g., Li et al., 2013; Hou et al., 2017a,b), compared with on-screen reading, text presentation on paper is more conducive to the construction of a mental map by the reader, thus, they can achieve better comprehension and a high degree of immersion, and will not easily fatigue. Printed texts present readers with fixed typography, chapter information, page numbers, corner frames, and blank spaces. During the process of flipping through a text on paper, rich kinesthetic feedback, such as visual perception and tactility, is also presented, and readers unconsciously know the physical location of specific information within a text and its spatial relationship to their location in the text as a whole. This ability to locate information is important for comprehension and recall because, when readers search for an object in their memory, they often locate it by recalling relevant background information cues (Chun and Jiang, 1998). In contrast, on-screen readers can only progress visual information (e.g., progress bars), and the lack of contextual information cues makes it difficult for readers to identify the location of specific information in the text. Moreover, scrolling may prevent readers from forming a coherent psychological representation. It is difficult for readers to remember the spatial location of a specific text section, since it changes position as the reader scrolls down.

Based on this, this study suggests that cognitive maps may play a crucial role in on-screen reading. The lack of sufficient background information and effective navigational cues in the presentation form of text on a screen hinders the construction of cognitive maps, thus leading to low reading performance.

Concerning question 2, in this study, for both paper and on-screen media, the comprehension and navigation scores under physical and verbal signals were significantly higher than those without signals, showing a signaling promotion effect. Paper reading has already been verified by many previous studies, and this study focused on how signals affect the construction of cognitive maps to achieve a promoting effect on on-screen reading. According to the existing research, this can be analyzed from two aspects of background information and navigation cues.

First, concerning the impact of signals on background information, rich background information not only helps the brain to process and encode textual content but also facilitates identification of the location and extraction of specific information (Chun and Jiang, 1998; Morineau et al., 2005). Although the background information during the reading process is not directly related to the content read, it provides cues about the structure of the text, so that a mental map with rich information about the entire text can be formed in the brain. Reading each page is akin to leaving a footprint on a map, which unknowingly equips readers with a clear spatial perception of what is being read. However, while on-screen reading, because of its constant presentation forms and indistinguishable external status, readers often find it difficult to localize a given part of the information within a text. At such a passage, the text is underlined, formatted in bold, and equipped with a marker of the organizing chart, which greatly enriches the background information, the text provides and helps to establish a “landmark” for the reader. During the reading process, the reader’s comparison and synthesis between the mark and the corresponding content, and each mark, form a relationship route. Based on this, a comprehensive psychological representation can be built that guides the comprehension and extraction of textual content.

Second, judging from the influence of signals on navigational cues, the text form presented on a screen lacks effective navigational cues, which is not conducive to the recall and review of textual content (e.g., Li et al., 2013; Hou et al., 2017b; Singer and Alexander, 2017). The cognitive map theory suggests that the reason why individuals can successfully navigate *via* spatial positioning is that they can use a map in their memory as a representation of space during navigation (i.e., to confirm the distance and direction between locations and flexibly plan routes; Weisberg and Newcombe, 2018). During the reading process, to complete the understanding, induction, and absorption of knowledge, readers will also locate information and switch between different areas within the text (Wästlund et al., 2008), i.e., will apply navigation of reading. During the learning phase, good navigation is helpful to construct cognitive maps, and promotes reader comprehension and text recall. During the information extraction phase, if the spatial location information of the target content is saved within cognitive maps, and if the connection route is smooth, such information can be quickly and accurately located. Otherwise, readers can only navigate *via* linear search, which not only consumes more cognitive resources but also greatly decreases performance. In the non-signaling condition of this study, comprehension and navigation scores of the paper reading group were significantly higher than those of the on-screen reading group. Moreover, the comprehension and navigation scores of the on-screen reading group were significantly improved after signaling, and were basically identical to those of the paper reading group. This indicated that signaling effectively compensates for the lack of navigational cues in on-screen texts, so that readers can also use cognitive maps to navigate when reading text on a screen, thus improving comprehension and navigational performance.

Regarding question 3, the results obtained by this study differ from the expectations, and also from the results of previous studies. For example, Su (2018) and others compared the impact of different signals on the paper reading performance of junior high school students, and found that an organizing chart can improve their performance better than text formatting *via* highlighting and underlining. Interestingly, this study found that when reading expository text on a screen, physical signaling exerts a stronger promotion effect on navigation and comprehension performance than verbal signaling. This difference is mainly derived from detailed recall questions rather than comprehension inference questions. Specifically, when readers are required to understand and grasp the main content of the text and reason with the central idea, the two signaling forms achieved the same promotion effect. When readers had to accurately recall detailed information and concepts, the on-screen reading score under physical signaling was significantly higher than that of verbal signaling.

The reason may be related to different signals that act on different phases of the construction of cognitive maps. For example, physical signaling acts on the first and second phases of cognitive map construction. On the one hand, physical signaling is directly embedded in the text by use of underlining, bold formatting, etc., which both enriches and clarifies the background information of the text. During reading, the words, phrases, or sentences with signals are then treated as landmarks. Landmark knowledge orients readers to navigate in an on-screen reading environment by visually highlighting the crucial information of the text. On the other hand, the highlighted landmark information *per se* has a specific logical relationship, which in combination with a landmark location, provides readers with interconnected navigational cues for the construction of the final situation model. This promotes the formation of routine knowledge during the second phase.

Verbal signaling (i.e., the presentation of an organizing chart, showing key items and their hierarchical relationships) acts on the formation of routine knowledge during the second phase. Previous studies mostly used reading software to embed organizing charts or navigational maps in reading materials, e.g., by presenting them on the side of a text page as a visual toolbar (Li et al., 2013; Sullivan and Puntambekar, 2015). Readers can automatically jump to the corresponding text content by clicking on a particular concept in the toolbar. This form of visual reading in the document leads to a closer connection between signals and information locations, and readers can use the organizing chart for real-time navigation and thus gradually improve the cognitive map in their memory. However, the organizing chart in this study is presented after the entire text, and thus, the signals and text are relatively independent and are not technically connected. While reading, readers may find it difficult to individually link the concepts of the topic in the organizing chart to the content of the text. This leads to the inability of the reader to identify the location of a headline corresponding to the original text and provide an answer based on the context when answering detailed questions.

This study only used behavioral experiments (a quantitative approach) to verify cognitive maps. It is possible to obtain more comprehensive findings by using a mixed methodological approach, for example, adding targeted interviews or open-ended survey questions. Future research should use a combination of quantitative and qualitative research methods. Besides, Given that eye-movement research can “directly” observe people’s cognitive processing during reading through eye movement indicators (Rayner, 1978, 1998), future studies should collect eye-movement information during on-screen reading to verification of the process of cognitive map construction, and investigate the impact of different forms of signaling on cognitive map construction during different phases by observing readers’ eye movement trajectories between different signaling contents to further explore the impact and functional mechanism of cognitive maps on text processing in the human brain.

The participants in this study were young college students majoring in engineering, who had high computer competency and familiarity with on-screen reading. Future research should also investigate a more diverse population to replicate the results, for example, age and major background may influence people’s reading comprehension. Moreover, the organizing chart used in this study is presented independently at the end of the text. If it were embedded in the text, readers could navigate the text in real-time, which would enable explorations of whether its promotion effect on on-screen reading will be equivalent to or even surpass that of underlining and bold formatting. At last, the reading materials in this study were short expository texts of about 1,200 words, which made it comparatively easier for readers to grasp the topic and structure of the text compared with longer texts. This may also be the reason why the promotion effect of verbal signaling in this research was not as pronounced as that of physical signaling. Future research should use the length of the reading material as a manipulatable variable to further unveil the signaling strategies suitable for on-screen reading, which will play an important role in the widespread promotion of on-screen reading in the future.

## Conclusion

This study showed that the use of signals can provide background information and navigational cues for on-screen reading, promote the construction of readers’ cognitive maps, and effectively improve their on-screen reading performance. Specifically, the following three results were found:

1. Reading expository text on computer screens was not as effective as reading these on paper.
2. Whether the text was presented on paper or screen, physical, and verbal signals of texts could help readers to navigate, construct cognitive maps, and improve their reading performance.
3. For on-screen reading, physical signaling exerted a stronger promoting effect than verbal signaling, and this difference was mainly derived from detailed recall questions rather than comprehension inference questions.

## Data Availability Statement

The raw data supporting the conclusions of this article will be made available by the authors, without undue reservation.

## Ethics Statement

The studies involving human participants were reviewed and approved by the Ethics Committee of Hunan Normal University in China. The patients/participants provided their written informed consent to participate in this study.

## Author Contributions

ZS and TT conceived and designed the experiments. LY and TT performed the experiments. TT analyzed the data. ZS and TT wrote the manuscript. All authors contributed to the article and approved the submitted version.

### Conflict of Interest

The authors declare that the research was conducted in the absence of any commercial or financial relationships that could be construed as a potential conflict of interest.
